# Haemorrhagic Cholecystitis: A Rare Form of Haemorrhagic Shock in a Patient With a Double Risk

**DOI:** 10.7759/cureus.107013

**Published:** 2026-04-14

**Authors:** Nicole Foreman, Ana Morais Alves, Ana Beatriz Machado, Catarina Sottomayor, Maria Manuel Moreira, Cristiana Paulo, José Manuel Pereira, José Artur Paiva

**Affiliations:** 1 Intensive Care Unit, Unidade Local de Saúde de São João, Porto, PRT; 2 Intensive Care Unit, Unidade Local de Saúde de Trás-os-Montes e Alto Douro, Vila Real, PRT; 3 Intensive Care Unit, Unidade Local de Saúde Entre Douro e Vouga, Santa Maria da Feira, PRT; 4 Medicine, Universidade do Porto, Porto, PRT

**Keywords:** anticoagulation, circulatory shock, haemophilia b, haemorrhagic cholecystitis, intensive care

## Abstract

Haemorrhagic cholecystitis is an uncommon condition with varied clinical presentations and several risk factors, such as anticoagulation, coagulopathy, malignancy, and trauma. We describe the clinical case of a 43-year-old man with two risk factors, anticoagulation and haemophilia B, who presented with atypical symptoms that progressed to shock. Diagnosis and therapeutic approach made this case particularly challenging. This condition should be a diagnosis to consider in a patient with relevant risk factors.

## Introduction

Haemorrhagic cholecystitis is a rare complication of acute cholecystitis, with a reported incidence of 7% [1-3], and there are only a few isolated cases described in the literature. The most common clinical presentations are cholecystitis and biliary colic, with haemorrhage being a rarer form of presentation [1]. Anticoagulation, coagulopathy, neoplasia, and trauma are some of the main well-established risk factors [1,2]. Preoperative diagnosis is uncommon, and no standardized diagnosis or management approach currently exists [1-3]. The treatment options range from a conservative strategy to urgent surgery, including multimodal approaches, combining interventional radiology or endoscopy with surgery. The latter is the most frequently reported strategy [3]. Haemophilia B is a hereditary bleeding disorder due to a deficiency of clotting factor IX. It has a wide bleeding phenotype from mild to severe forms, which is related to the level of IX factor [4]. In these patients, haemorrhagic events are a serious concern and require an individualized and multidisciplinary treatment approach.

We present a case of a haemorrhagic and distributive shock due to haemorrhagic cholecystitis in a patient with type B haemophilia and anticoagulation. Several features make this case remarkable, including its rarity and atypical clinical presentation. More importantly, to our knowledge, it represents the first case reported in the literature with the coexistence of two bleeding risk factors, haemophilia B and anticoagulation.

## Case presentation

**Figure 1:**
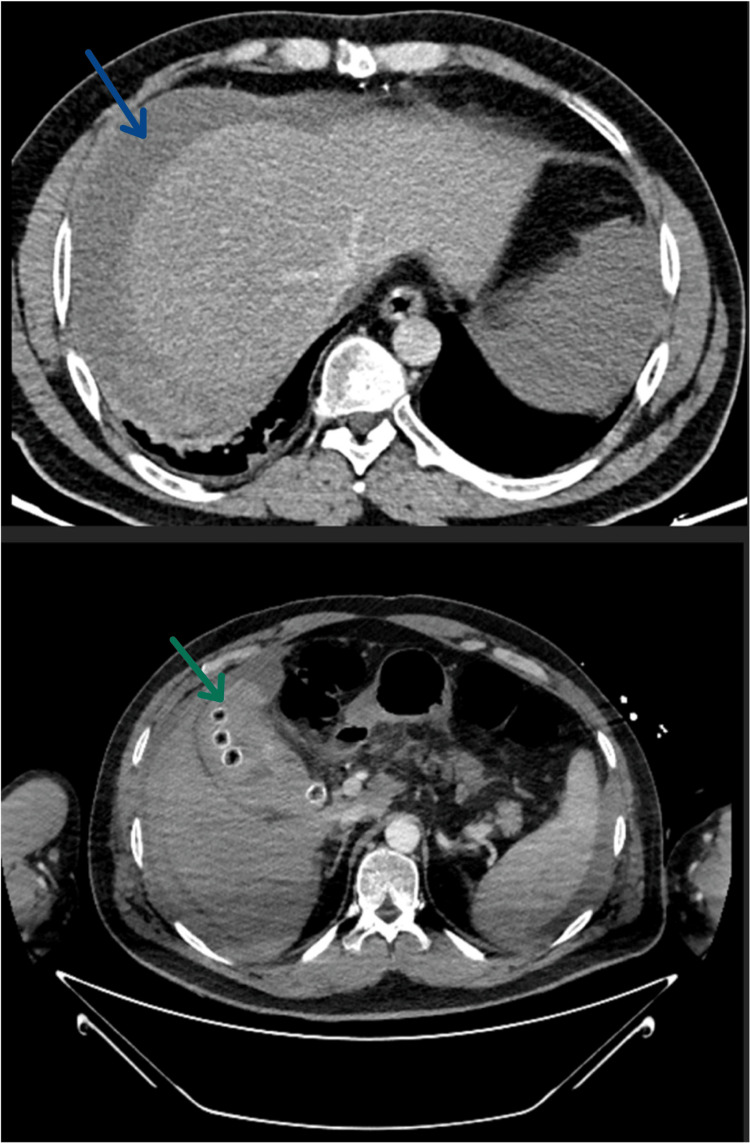
Abdominopelvic computed tomography Abdominopelvic computed tomography scan with angiography demonstrating an atypical gallbladder (green arrow) and a hemoperitoneum (blue arrow)

A 43-year-old man presented to the emergency room with intermittent pain in the right iliac fossa, radiating to the right shoulder for three days, associated with nausea and vomiting, without fever or other symptoms. His past medical history included mild type B haemophilia (factor IX: 30%) under on-demand supplementation and mechanical aortic and mitral valve prostheses, placed following previous endocarditis, for which he was under anticoagulation with warfarin. The physical examination at the emergency department revealed tenderness in the right upper and lower abdominal quadrants. During the first clinical observation, the patient's clinical condition deteriorated, progressing to circulatory shock, with hypotension and hyperlactacidaemia. The blood results revealed acute anaemia, a supratherapeutic international normalized ratio (INR), a subtherapeutic factor IX level, and an elevated C-reactive protein and leucocyte count, as represented in Table 1. Abdominopelvic computed tomography (CT) scan with angiography revealed an atypical gallbladder image (Figure 1), with marked distension and hyperdense intraluminal content associated with gallstones. Moderate volume hemoperitoneum was also observed. In addition to transfusion support with packed red blood cells, anticoagulation was reversed using vitamin K, prothrombin complex concentrate, and factor IX. Besides the cardiovascular and haematological dysfunction, the patient also had a mild hypoxemic respiratory insufficiency. There were no other major dysfunctions. The patient was stabilized and admitted to the intensive care department for close monitoring.

**Table 1:** Relevant laboratory results INR: international normalized ratio

Parameters	Reference values	Results at admission
Haemoglobin (g/dL)	13-18	7.4 (basal 13)
INR	2-3 (therapeutic value)	3.3
Factor IX level (U/mL)	0.7-1.2	0.45
Leucocytes (×10^9^/L)	4-11	11.6
C-reactive protein (mg/L)	<3	116
Lactate (mmol/L)	<2	3.68

Due to the high bleeding risk, surgical intervention was initially postponed, and an approach by interventional radiology was considered. However, within the first hours of admission, the patient's condition worsened due to macrocirculatory dysfunction. A CT angiography was repeated. Although no active bleeding was identified, haemorrhagic cholecystitis was strongly suspected, and urgent laparoscopic cholecystectomy was performed. Macroscopically, the gallbladder was perforated and showed signs of inflammation, and for this reason, empirical antibiotic therapy with metronidazole and ceftriaxone was prescribed. The histopathological results of the gallbladder revealed signs compatible with acute on chronic cholecystitis. There were no microbiological isolations.

Management of anticoagulation was challenging and required close collaboration with the immunohemotherapy department. During the postoperative period, the patient maintained haemorrhagic drainage originating from the gallbladder bed. Despite ongoing low-flow drainage, angiography evaluation showed no active bleeding, and there were no signs of haemodynamic instability or haemoglobin decline. This condition was managed conservatively. Simultaneously, reintroduction of anticoagulation was mandatory due to the prosthetic mechanical heart valves in order to prevent thrombotic complications. Anticoagulation was restarted with unfractionated heparin under anti-Xa monitoring. Factor IX levels were measured daily, maintaining a higher target threshold (>60%) due to bleeding risk. Achieving balance between anticoagulation and adequate factor IX levels was critical. The patient experienced a favourable clinical outcome without thrombotic events and was discharged to the ward after 16 days of intensive care unit stay.

## Discussion

Several features make this case noteworthy. First is the rarity of haemorrhagic cholecystitis. According to the literature, its incidence is low [3], with a recent study reporting an incidence as low as 0.55% [1]. Second is the atypical clinical presentation. The spectrum of clinical manifestations is wide [3]. Our patient presented with referred pain and progressed to shock, the most severe clinical presentation within this spectrum. According to the literature, atypical presentation and progression to shock are uncommon, with an estimated incidence of approximately 17% [3]. Nevertheless, most reported shock cases initially present with mild symptoms that later progress to circulatory shock [3], consistent with our case. Only a limited number of cases describing haemorrhagic shock have been reported in the literature [5-8]. In addition to haemorrhagic shock, our case also had a distributive shock component associated with the gallbladder inflammation and infection. The pathophysiology of this disease is not well-defined in the literature [1,2]. In this case, the most plausible mechanism involves inflammation and mucosal erosion caused by gallstones, in a patient with a double haemorrhage risk, leading to intraluminal bleeding, gallbladder distension, perforation, and subsequent hemoperitoneum [2]. Third, this case is distinguished by the presence of two haemorrhagic risk factors: haemophilia and therapeutic anticoagulation. Few cases of haemorrhagic cholecystitis are described in the literature, with haemophilia [9,10] or anticoagulation/antiaggregation [11-13] as a common and only risk factor. To the best of our knowledge, this is the first case reported describing the coexistence of both risk factors.

Furthermore, this case presented other challenges. According to the literature, while cholecystectomy was previously the most common treatment reported in clinical cases, a recent review shows that the most frequent treatment modality now involves a multimodal approach [3]. This typically includes interventional radiology or endoscopy, followed by urgent cholecystectomy [3]. The best treatment strategy relies on the individual clinical assessment of the patient, including the clinical presentation severity, risk factors, and the local anatomical complexity [3]. Our case represented an additional challenge because of the high risk of bleeding. At first, a more conservative approach was considered with interventional radiology, but due to the clinical deterioration, an urgent cholecystectomy was required.

Regarding the antibiotic therapy, although rare, it may be considered alone, as a conservative treatment strategy in haemodynamically stable patients [3]. Additionally, antibiotic therapy may play a role as an adjunctive treatment, concerning that haemorrhagic cholecystitis is usually a clinical manifestation of acute cholecystitis. In the absence of risk factors for multidrug-resistant microorganisms, ceftriaxone and metronidazole are the usual empirical recommended strategy [1]. This case of haemorrhagic cholecystitis revealed signs consistent with gallbladder inflammation and infection, as well as evolution to shock with a distributive component; therefore, antibiotic therapy was started as an adjunctive treatment strategy.

Thromboembolism and prosthetic valve thrombosis are two major concerns regarding mechanical valve prosthesis. Anticoagulation is imperative to reduce the risk. The resumption of anticoagulation and timing must consider the risks and benefits on an individual and multidisciplinary basis assessment. This case represented a challenge because of ongoing low-flow hematic drainage, risk of bleeding due to haemophilia, and anticoagulation requirement due to the high risk of mechanical heart valve thrombosis. Haemodynamic stability, daily amount of hematic flow drainage, and haemoglobin and factor IX serum levels represented the main factors considered in the decision and timing of anticoagulation restart. Anticoagulation with unfractionated heparin was initiated under anti-Xa close monitoring. Anticoagulation monitoring with anti-Xa is the preferred option in patients with a prolonged activated partial thromboplastin time at baseline, for example, in haemophilia [14].

We believe this case is important for guiding future management of similar clinical scenarios.

## Conclusions

This case represented a significant challenge due to the rarity of haemorrhagic cholecystitis occurring in a patient with a double bleeding risk (hereditary coagulopathy and therapeutic anticoagulation) requiring individualized management. Although rare, haemorrhagic cholecystitis should be considered in the differential diagnosis of acute cholecystitis in a patient with bleeding risk factors and evidence of haemorrhage. An individualized assessment regarding the anticoagulation reversal, the best treatment strategy, and the timing of anticoagulation resumption is recommended in such patients.
